# Prognostic Factors for Mortality in Catheter-Related Bloodstream Infections Among Hemodialysis Patients: A Prospective Single-Center Study

**DOI:** 10.3390/medicina61122205

**Published:** 2025-12-12

**Authors:** Rukiye Inan Sarıkaya, Omer Karaşahin

**Affiliations:** 1 Department of Infectious Diseases and Clinical Microbiology, Faculty of Medicine, Balıkesir University, 10145 Balıkesir, Türkiye; 2 Department of Infectious Diseases and Clinical Microbiology, Health Sciences University Erzurum Regional Education and Research Hospital, 25240 Erzurum, Türkiye

**Keywords:** catheter, hemodialysis, infection, mortality, prognosis

## Abstract

*Background and Objectives*: Catheter-related bloodstream infection (CRBSI) is a major life-threatening complication among patients undergoing hemodialysis (HD) through central venous catheters. This study was performed to determine mortality risk factors in HD patients with CRBSI. *Materials and Methods*: Data were collected prospectively from patients with CRBSI using central venous catheters as HD access between November 2022 and November 2023. A total of 60 patients were evaluated with respect to age, sex, catheter dwell time, insertion site, comorbidities, and a range of clinical findings. Demographic and clinical characteristics were compared between survivors and non-survivors. A *p*-value < 0.05 was considered statistically significant. Risk factors were assessed through univariate and multivariate regression analyses. *Results*: The median age of the patients was 63 years, and 56.9% were male. The in-hospital mortality rate among the HD patients with CRBSI was 15%. Gram-positive microorganisms were responsible for 55.6% of the cases resulting in mortality. The following factors were associated with mortality: multidrug-resistant (MDR) microorganisms, the presence of a non-tunneled catheter, concomitant pyocystitis, elevated C-reactive protein, qSOFA ≥ 2, and altered consciousness. In multivariate logistic regression, MDR microorganisms, concomitant pyocystitis, and qSOFA ≥ 2 remained significant independent predictors of mortality. *Conclusions*: Our findings show that the above factors may be useful in predicting mortality in HD patients with CRBSI. Awareness of these factors and prompt intervention can help reduce mortality.

## 1. Introduction

Hemodialysis (HD) is the most commonly used renal replacement therapy for patients with end-stage renal disease. Effective and safe HD requires adequate vascular access. In clinical practice, the available vascular access options include arteriovenous fistulas (AVF), arteriovenous grafts, and tunneled or non-tunneled central venous catheters (CVC). Although current guidelines recommend AVF as the first-line access for long-term HD, AVF creation may not be feasible in certain circumstances, such as prolonged maturation time, advanced age, atherosclerotic vascular disease, congestive heart failure, or limited life expectancy. Therefore, a considerable proportion of patients—particularly those newly starting HD—initiate treatment with either tunneled or non-tunneled CVCs. It has been reported that approximately 30% of patients continue long-term HD with CVC access [1]. Despite their practical advantages, CVCs are associated with serious complications such as venous thrombosis, central venous stenosis, mechanical problems, and, most importantly, catheter-related bloodstream infections (CRBSI). Among these complications, CRBSI stands out as the one with the greatest impact on mortality and morbidity. Indeed, infections represent the second leading cause of death after cardiovascular events in HD patients, and this elevated risk is strongly driven by catheter-related infections. Infections are the second leading cause of death after cardiovascular events in hemodialysis (HD) patients, and catheter use is the most important determinant of this risk [2,3,4]. Bloodstream infections (BSIs) are particularly common in HD patients using central venous catheters, depending on the type of vascular access, the incidence of BSI in HD patients has been reported to range widely from 0.5 to 27.1 episodes per 100 patient-months [5]. HD patients are at an extremely high risk of sepsis-related mortality compared with the general population due to impaired immune function and prolonged exposure to vascular interventions, and the annual sepsis mortality rate is 50–100 times higher than in the general population [6].

In catheter-related bloodstream infection, Gram-positive bacteria, particularly *Staphylococcus aureus* and coagulase-negative staphylococci, predominate etiologically, whereas Gram-negative pathogens also account for a considerable proportion of cases [2,3]. It is well known that *S. aureus* bacteremia in HD patients leads to serious complications such as endocarditis and metastatic infections and is associated with higher mortality than bacteremia caused by other pathogens. Another adverse prognostic indicator in these patients is the development of septic shock or the presence of polymicrobial infection [5].

Permanent AVF is the safest form of vascular access in HD patients, whereas the use of central venous catheters significantly increases the risk of infection and mortality. In particular, patients dialyzed through non-tunneled temporary catheters experience higher rates of infection and sepsis-related adverse outcomes. In a recent cohort study, 90% of HD patients who died in hospital were reported to have a dialysis catheter in place, most often a non-tunneled catheter [7]. Therefore, catheter-related bloodstream infections remain an important cause of mortality in HD patients, and elucidating prognostic factors in this context is of substantial clinical importance [5].

Previous studies [8,9,10] have reported the risk factors, prognosis, and the spectrum of pathogens associated with CRBSI. Mortality studies [7,11,12] have focused more on risk factors for adverse outcomes (recurrent CRBSI, endocarditis, and metastatic infection) and causes of mortality in HD patients. In contrast, there are relatively few reports [13,14,15,16] detailing the distribution of causative pathogens specifically in HD patients with CRBSI and the risk factors for CRBSI-related and in-hospital mortality. Identifying potential risk factors that predict in-hospital mortality may help reduce mortality by informing the development of effective prevention strategies and treatment guidelines. Therefore, in this prospective single-center study, we aimed to determine prognostic factors that predict in-hospital mortality in HD patients who develop CRBSI.

## 2. Materials and Methods

### 2.1. The Study Design and Population

This was a prospective observational study investigating prognostic factors in HD patients diagnosed with CRBSI at a tertiary care center. The study was approved by the Erzurum Regional Training and Research Hospital Ethics Committee (Decision No.: Erzurum BEAH KAEK 2022/17-163; date: 7 November 2022). The research was conducted between November 2022 and November 2023 in a tertiary health care institution. The study population consisted of patients receiving HD via tunneled or non-tunneled central venous catheters (CVCs) who were diagnosed with CRBSI. Adults aged ≥18 years who were receiving chronic HD and were dialyzed via temporary or permanent CVCs were included in the study. Secondary bacteremia attributable to another focus of infection apart from the dialysis catheter, cases with only local catheter infection and negative blood cultures, catheter colonization, and patients with incomplete data were excluded. Patients were followed prospectively throughout the period of hospitalization for treatment of CRBSI. The study was conducted in accordance with the principles of the Declaration of Helsinki. A total of 60 HD patients (age range: 28–92 years) were included. Written informed consent was obtained from all participants

### 2.2. Data Collection

Detailed medical histories were obtained from all participants. Demographic characteristics, comorbidities, duration of HD, catheter type used, and catheter dwell time were recorded. At the time of BSI onset, clinical findings at presentation, vital signs, and the quick Sequential Organ Failure Assessment (qSOFA) score were documented. Initial laboratory parameters assessed included hemoglobin (g/dL), leukocyte, neutrophil, lymphocyte, and platelet counts (×10^9^/L), alanine aminotransferase (ALT) (U/L), aspartate aminotransferase (AST) (U/L), creatinine (mg/dL), blood urea nitrogen (BUN) (mg/dL), glucose (mg/dL), albumin (g/dL), C-reactive protein (CRP) (mg/L), international normalized ratio (INR), D-dimer (ng/mL), ferritin (ng/mL), and procalcitonin (ng/mL).

At least two sets of blood cultures were obtained from all patients using appropriate techniques; the species of isolated microorganisms and their antibiotic susceptibility profiles were recorded. The presence of other infection foci (e.g., pyocystitis, cholangitis, pneumonia) accompanying the bacteremia was investigated. During the course of treatment, necessary consultations (e.g., evaluation for endocarditis by echocardiography) were performed, and complications such as infective endocarditis were documented. The need for intensive care and in-hospital mortality outcomes were prospectively followed.

### 2.3. Definitions

Catheter-related bloodstream infection was defined according to the 2009 IDSA Clinical Practice Guidelines. In all cases, paired blood cultures were obtained simultaneously from the catheter lumen and a peripheral vein. The primary microbiological criterion for diagnosis was the differential time to positivity (DTP). Growth of the same microorganism from the catheter-lumen sample ≥ 2 h earlier than the peripheral sample was considered indicative of CRBSI. Catheter-tip cultures were performed only in patients whose catheters were removed for clinical reasons. In patients in whom the catheter was not removed, the diagnosis was established based on compatible clinical findings, a positive blood culture fulfilling the DTP criterion, and exclusion of alternative infectious sources [17]. All catheter insertions were performed under maximum barrier precautions in accordance with our tertiary-care center protocols, including full sterile draping, sterile gown, mask, cap, sterile gloves, and wide-area skin antisepsis. Management of suspected CRBSI followed our institutional protocol based on international guidelines. In patients with severely limited vascular access options or high bleeding risk, however, temporary catheter retention was allowed at the discretion of the treating infectious diseases specialist.

The quick Sequential Organ Failure Assessment (qSOFA) score was calculated at baseline as a prognostic tool. This score comprises three parameters: systolic blood pressure ≤ 100 mmHg, respiratory rate (RR) ≥ 22/min, and Glasgow Coma Scale score < 15, with the presence of ≥2 criteria indicating severe infection [18].

Multidrug resistance (MDR) was defined as acquired resistance to at least one antimicrobial agent in three or more drug categories [19].

Pyocystis is a rare but clinically significant lower urinary tract infection characterized by the accumulation of purulent infected material within the bladder lumen, typically occurring in a defunctionalized bladder in anuric end-stage renal failure patients [20].

CRBSI-related mortality was defined as death occurring during the same hospitalization in which CRBSI was diagnosed by the clinical team. The primary outcome measure was in-hospital mortality, defined as death during the index hospitalization, with comparisons made between survivors and non-survivors. In this cohort, all deaths that occurred during the index hospitalization were clinically assessed by the treating team and judged to be directly attributable to CRBSI and its infectious complications. No unrelated causes of death were identified. Moreover, all deaths occurred within 30 days of CRBSI diagnosis; therefore, 30-day mortality and in-hospital mortality were identical (15%; 9/60), and a separate 30-day endpoint was not defined.

### 2.4. Statistical Analysis

The data obtained were analyzed using SPSS version 25.0 (IBM Corp., Armonk, NY, USA). Continuous variables were presented as median and interquartile range (IQR) or mean ± standard deviation, as appropriate, and categorical variables as number and percentage. For comparisons, continuous data were analyzed using the Mann–Whitney U test or Student’s *t*-test, and categorical data were compared using the chi-square test or Fisher’s exact test. To identify risk factors affecting in-hospital mortality, univariate logistic regression analyses were first performed for each variable. Variables with *p* < 0.05 in univariate analysis were then entered into a multivariate logistic regression model using a backward stepwise procedure. Factors independently associated with mortality in the multivariable analysis were determined, and the odds ratio (OR) and 95% confidence interval (CI) for each were calculated. In addition, receiver operating characteristic (ROC) curve analysis was performed to evaluate the predictive power of independent risk factors for mortality, and corresponding sensitivity and specificity values were calculated. A *p* value < 0.05 was considered statistically significant for all tests.

## 3. Results

### 3.1. Patient Baseline Characteristics

The median age of the 60 HD patients included in the study was 64.0 years (min–max, 28–92), and 56.7% were male (Table 1). The median length of hospital stay was 19.5 days (IQR, 14–28 days). Infective endocarditis was diagnosed in 7 patients (11.6%); right-sided (tricuspid valve) endocarditis was identified in 6 cases and left-sided endocarditis in 1 case. During follow-up, 10 patients (16.7%) were transferred to the intensive care unit. Overall, 9 patients (15.0%) died during the index hospitalization, whereas the remaining 51 patients recovered with treatment and were discharged.

### 3.2. Microbiological Findings

**Figure 1:**
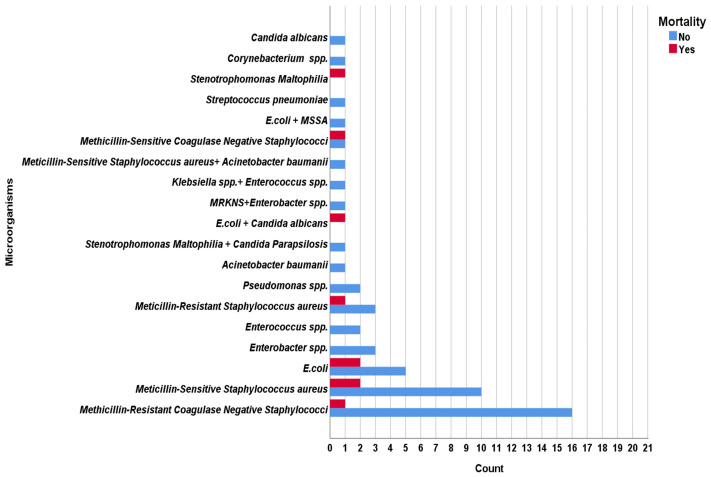
Distribution of causative microorganisms stratified by survival status.

Causative microorganisms and their resistance profiles, based on blood culture results obtained from all cases, are shown in Figure 1 stratified by survival status. Overall, the most frequently isolated pathogens were methicillin-resistant coagulase-negative Staphylococcus species, *S. aureus*, and *E. coli*. Of all positive cultures, 66% were due to Gram-positive bacteria and 33% to Gram-negative bacteria; Candida species were isolated in 1 patient. From a microbiological standpoint, infections caused by multidrug-resistant (MDR) pathogens were found to be significantly associated with a fatal course. In 8 of the patients who died (88.9%), the causative organism was MDR, whereas the proportion of MDR pathogens among survivors was 37.3% (*p* = 0.005). Among the *Staphylococcus aureus* isolates, 4 cases were methicillin-resistant, and the remainder were methicillin-susceptible; only one of the four MRSA infections resulted in death. Polymicrobial infections were present in 1 non-survivor (11.1%) and in 5 survivors (9.8%); although numbers were small and the difference was not statistically significant, polymicrobial infections occurred slightly more frequently in the mortality group. The Gram-positive versus Gram-negative status of the isolated pathogen did not, by itself, emerge as a determinant of mortality (proportion of Gram-negative pathogens: 44.4% in non-survivors vs. 23.5% in survivors; *p* = 0.328). Candida infection was observed in only 1 case (in the survivor group) and was therefore not suitable for meaningful analysis in relation to mortality.

### 3.3. Identification of Prognostic Factors

Baseline characteristics and clinical findings of patients who died are summarized in Table 2, Table 3 and Table 4. Demographic features, including age and sex distribution, did not differ significantly between survivors and non-survivors (median age 64 vs. 63 years, *p* = 0.330). Similarly, the number of comorbid conditions and Charlson comorbidity index scores were not significantly associated with mortality (all *p* > 0.05). However, several critical clinical signs and risk factors were significantly more frequent in patients who died. Patients presenting with confusion had a distinctly worse prognosis: 66.7% of non-survivors had confusion at presentation, compared with 17.6% of survivors (*p* = 0.005). Likewise, an initial qSOFA score ≥ 2 was associated with an increased risk of death (present in 55.6% of non-survivors vs. 19.6% of survivors; *p* = 0.022). Although individual vital sign abnormalities such as hypotension, fever, and tachycardia were observed more often among non-survivors, these differences did not reach statistical significance when evaluated separately. In contrast, when considered collectively through the qSOFA score—reflecting dysfunction across multiple organ systems—a higher score emerged as a distinct prognostic discriminator.

Approximately one-fifth of patients (23.3%) had an additional, concurrent infectious focus other than catheter-related bacteremia. In particular, the presence of pyocystitis was strongly associated with a fatal outcome: 55.6% of non-survivors had concomitant pyocystitis, compared with only 11.8% of survivors (*p* = 0.007). Overall, the presence of any additional infectious focus was documented in 66.7% of patients who died and 15.7% of those who survived (*p* = 0.003). Among secondary infectious foci, pyocystitis was the most frequent; the isolated pathogens in pyocystitis cases were *Escherichia coli* (6 cases), *Klebsiella pneumoniae* (2 cases), *Enterococcus faecium* (2 cases), and methicillin-susceptible *S. aureus* (MSSA) (1 case).

When laboratory parameters at presentation were compared, CRP level was found to be associated with mortality. The median CRP level was 203 mg/L in non-survivors and 110 mg/L in survivors (*p* = 0.042). Among other inflammatory markers, procalcitonin levels tended to be higher in the mortality group but showed wide variability (median 9.1 ng/mL vs. 3.7 ng/mL, *p* = 0.872). No significant differences were observed between survivors and non-survivors with respect to leukocyte count, neutrophil and lymphocyte counts, hemoglobin, platelet count, renal function tests, liver enzymes, or coagulation parameters (all *p* > 0.05). A noteworthy finding among non-survivors was a slightly lower albumin level (median 3.5 g/dL vs. 3.6 g/dL); however, this difference did not reach statistical significance (*p* = 0.282).

When mortality rates were analyzed according to the type of dialysis catheter used, non-tunneled (temporary) catheters were associated with substantially higher mortality. At the time of the event, 66.7% of non-survivors were dialyzed via a temporary catheter, whereas only 17.6% of survivors had a non-tunneled catheter in place (*p* = 0.005). Conversely, tunneled (permanent) catheters were present in 33.3% of non-survivors and 82.4% of survivors. The anatomical site of catheter insertion (subclavian, jugular, femoral) was not significantly associated with mortality (*p* = 0.650); however, 3 of the 9 patients who died had a femoral catheter, and femoral access sites were numerically associated with a higher mortality rate (33.3% vs. 23.5%), without reaching statistical significance. The duration of catheter placement (longer vs. shorter indwelling time) was not an independent predictor of mortality (*p* > 0.354). Similarly, removal of the catheter within the first 48 h after detection of infection did not significantly affect survival (early catheter removal in 11.1% of non-survivors vs. 19.6% of survivors, *p* = 0.475). To further explore the impact of the early haemodialysis period, catheter dwell time at the onset of CRBSI was categorised as ≤6 months (n = 28) versus >6 months (n = 32). In-hospital mortality tended to be higher in patients with a catheter in place for ≤6 months (21.4% vs. 9.4%; *p* = 0.192), whereas the median length of hospital stay was similar between groups (21 [IQR 12–30] vs. 17 [14–25] days; *p* = 0.462). Notably, MDR pathogens were significantly more frequent in the ≤6 month group (60.7% vs. 31.3%; *p* = 0.021), suggesting a higher burden of MDR CRBSI during the early dialysis period.

In univariate analysis, the following factors were significantly associated with mortality: infection with an MDR pathogen (OR, 13.47; *p* = 0.018), use of a non-tunneled catheter (OR, 9.33; *p* = 0.005), presence of concomitant pyocystitis (OR, 10.75; *p* = 0.003), confusion at presentation (OR, 9.33; *p* = 0.005), qSOFA score ≥ 2 (OR, 5.13; *p* = 0.031), and elevated CRP level (OR, 1.007 per 1 mg/L increase; *p* = 0.075). These variables were entered into a multivariable logistic regression model to control for potential interactions. Multivariable analysis identified three independent prognostic factors: infection with an MDR microorganism, the presence of an additional infectious focus such as pyocystitis, and qSOFA ≥ 2 (Table 5). CRBSI due to an MDR bacterium increased the risk of mortality by approximately 14.85-fold compared with infections caused by susceptible organisms (95% CI, 1.28–172.58; *p* = 0.031). Similarly, in cases with concomitant pyocystitis, the risk of death was 13.06 times higher than in those without pyocystitis (95% CI, 1.70–100.23; *p* = 0.013). Patients with an initial qSOFA score ≥ 2 had an 8.76-fold higher risk of mortality compared with those with qSOFA < 2 (95% CI, 1.18–65.14; *p* = 0.034). Other factors—non-tunneled catheter use, confusion, and elevated CRP—did not retain independent significance in the multivariable model; notably, confusion appears to have had its effect subsumed by qSOFA, of which it is a component.

**Figure 2:**
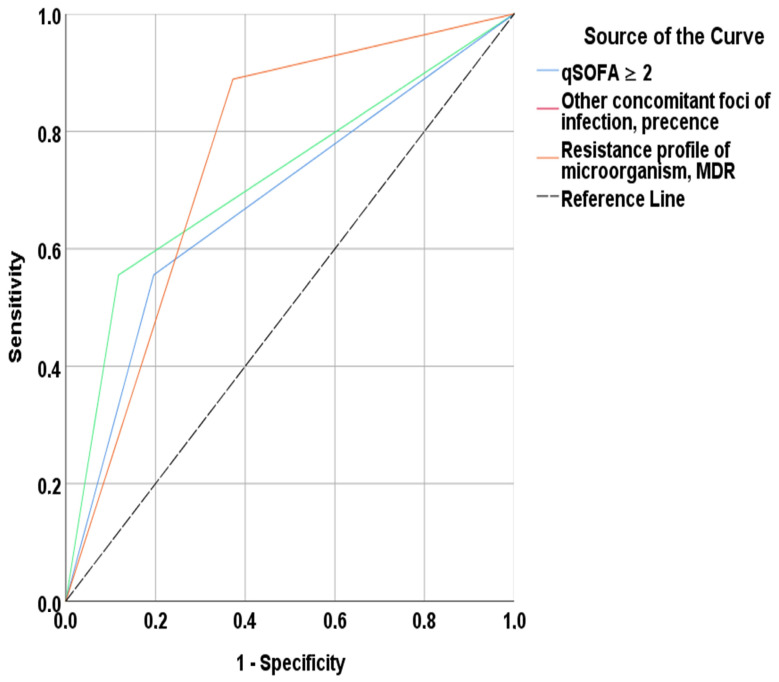
ROC curves for independent predictors of in-hospital mortality.

To evaluate the performance of these independent risk factors in predicting mortality, receiver operating characteristic (ROC) curves were constructed (Figure 2). For MDR pathogen status, the area under the curve (AUC) was 0.758 (95% CI, 0.606–0.911; *p* = 0.014), with a sensitivity of 88.9% and a specificity of 62.7%. The AUC for the qSOFA ≥ 2 criterion was 0.709 (95% CI, 0.503–0.916; *p* = 0.047), yielding a sensitivity of 55.6% and a specificity of 82.2%. The presence of a concomitant infectious focus had an AUC of 0.755 (95% CI, 0.563–0.947; *p* = 0.015), with a sensitivity of 66.0% and a specificity of 84.3% for predicting mortality.

## 4. Discussion

In this prospective single-center study, the in-hospital mortality rate among HD patients with CRBSI was 15%. These findings are consistent with the 8–20% mortality range reported in previous studies [16,21,22,23]. In contrast, some cohorts have reported substantially lower mortality rates; Shahar et al. found a mortality rate of only approximately 1%. This low rate is thought to stem from methodological and clinical differences, such as the inclusion of a less severely ill patient population, the early initiation of broad-spectrum empirical antibiotic therapy, and the predominant use of tunneled catheters [15]. In our cohort, several factors were associated with mortality on univariate analysis, including infection with MDR organisms, use of a non-tunneled catheter, concomitant pyocystitis, elevated CRP, qSOFA ≥ 2, and altered mental status at presentation. However, in multivariate analysis only three emerged as independent predictors of mortality: MDR pathogen, concomitant pyocystitis, and qSOFA ≥ 2. Other variables–notably high CRP, confusion, and catheter type–lost their independent significance, likely due to correlation with overall illness severity and the limited sample size.

Timely identification of the causative microorganism and its antimicrobial susceptibility is critical for guiding therapy in CRBSI. In our study, Gram-positive bacteria accounted for 55.6% of the infections in patients who died, while Gram-negative bacteria caused 44.4% of the fatal cases. This pathogen distribution is comparable to that reported in other hemodialysis cohorts [8]. *S. aureus* is a well-known cause of bacteremia in HD patients that can lead to severe complications such as endocarditis and osteomyelitis, especially in the case of methicillin-resistant strains. Indeed, previous studies indicate that *S. aureus* bacteremia in dialysis patients is associated with higher rates of metastatic infection and morbidity compared to other pathogens [12,14]. However, in our cohort *S. aureus* infections did not result in significantly higher mortality than infections caused by other organisms. This finding may be attributed to the fact that most cases were caused by MSSA infections, in which appropriate empirical antimicrobial therapy is usually not delayed. In addition, our unit, empirical therapy for suspected CRBSI routinely consisted of a broad-spectrum β-lactam combined with an anti-MRSA agent, and all MRSA cases were covered by a glycopeptide from the start; therefore, early appropriate empirical antibiotic therapy was achieved in almost all patients and did not function as a discriminative prognostic factor in this cohort. Nevertheless, the predominance of *S. aureus* as the causative pathogen among CRBSI cases complicated by endocarditis underscores its propensity to cause metastatic infectious foci and its potential for poor clinical outcomes.

One of the most striking findings was the strong impact of antimicrobial resistance on outcomes. In fact, the presence of an MDR pathogen increased the odds of in-hospital death by approximately an order of magnitude (15-fold) in our analysis. This aligns with observations that antibiotic resistance portends worse outcomes in bloodstream infections generally. The prevalence of MDR pathogens in catheter-related infections among hemodialysis patients varies considerably across the literature, with some series reporting rates around 12–13%, while others have documented rates of 36% or even higher [8,9,10]. In our series, 37.3% of isolates from survivors were MDR, whereas an alarming 88.9% of isolates from non-survivors were MDR, highlighting why resistance is such a critical determinant of prognosis. MDR Pseudomonas strains, although not typically highly virulent, can lead to adverse clinical outcomes due to their strong biofilm-forming capacity, intrinsic resistance mechanisms, and poor response to empirical therapy. In our cohort, the association between MDR organisms and mortality is consistent with previous reports emphasizing the prognostic significance of biofilm-producing and difficult-to-treat pathogens in CRBSI [24]. Possible reasons for the poor outcomes with resistant infections include the greater virulence of these organisms and delays in administering effective therapy. Infections like MRSA or those caused by ESBL-producing Gram-negatives may not respond to standard empiric antibiotics, allowing the illness to progress unchecked until appropriate therapy is initiated. Therefore, early and appropriate antibiotic treatment–promptly tailored based on culture results–is vital in managing HD patients with suspected CRBSI. Our findings underscore the need for stringent infection control measures in dialysis units and prudent antibiotic management to prevent and combat MDR infections [25].

Another notable observation was the role of vascular access. Non-tunneled dialysis catheters were used in a large proportion of the patients who died. While catheter type did not remain an independent predictor in the multivariable model, univariate analysis indicated worse outcomes with temporary catheters. This is consistent with other reports that link catheter dependence to higher mortality risk in dialysis patients [21]. A recent study found that 65% of hospitalized HD patients who died were using a non-tunneled catheter at the time [7]. Non-tunneled catheters carry the highest infection risk and are frequently placed for emergent dialysis initiation in already fragile patients, which may partly explain their association with poor outcomes. Moreover, such catheters typically provide lower blood flow rates and less efficient dialysis than tunneled catheters, potentially impacting adequacy and the patient’s resilience [26]. Our exploratory analysis of catheter dwell time indicated that MDR pathogens were significantly more common when the catheter had been in place for ≤6 months, whereas differences in mortality did not reach statistical significance. This pattern is consistent with the concept that the early haemodialysis ‘induction’ period, marked by uremic immunosuppression and malnutrition, may carry an increased risk for MDR CRBSI, although larger cohorts are needed to confirm this association [27]. In our cohort, early catheter removal within 48 h after CRBSI diagnosis was achieved in only 18.3% of episodes, reflecting the frequent lack of alternative vascular access in this end-stage renal disease population. This practical constraint may have limited the potential benefit of catheter withdrawal and should be considered when interpreting our findings. These findings emphasize that catheter use should be minimized in HD patients; whenever possible, permanent vascular access (AVF or graft) should be established early to reduce the reliance on temporary catheters and thereby decrease infection-related morbidity and mortality.

We also found that the presence of an additional infection focus concurrent with the catheter infection significantly increased the risk of death. Chronic kidney disease and dialysis patients are known to experience a high incidence of urogenital infections, and urosepsis in this population has been associated with high mortality [28,29]. In our cohort, concomitant pyocystitis (a purulent urinary tract infection) was particularly ominous–its presence increased the odds of in-hospital death by approximately thirteen-fold. Consistent with this, Prabhahar et al. observed that fatal cases in CKD patients often involved multi-regional infections rather than an isolated bacteremia [30]. Notably, *E. coli* was the most common pathogen in cases of pyocystitis accompanying CRBSI in our series, which aligns with the typical predominance of Gram-negative uropathogens reported in HD patients [29,30]. The poor prognosis in patients with multiple simultaneous infections is likely due to the higher overall microbial burden and an overwhelmed host immune response, as well as potential delays or difficulties in recognizing and treating all infection sites promptly [31]. Thus, when an HD patient is diagnosed with a catheter infection, it is critical to evaluate for other potential infection foci; if multiple infections are present, aggressive combined management and close monitoring are warranted.

In our study, a qSOFA score ≥ 2 emerged as a strong predictor of mortality. qSOFA is a simple scoring tool proposed in the Sepsis-3 definitions and is based on mental status, respiratory rate, and blood pressure parameters. In patients with severe infections, qSOFA ≥ 2 indicates a high risk of mortality [32,33]. Indeed, in one emergency department cohort, the 28-day mortality rate was reported as 26.4% among patients presenting with suspected infection and a triage qSOFA ≥ 2, compared with 11.7% in those with qSOFA < 2 [33]. Our findings similarly demonstrate the prognostic value of qSOFA in hemodialysis patients. A qSOFA score ≥ 2 essentially reflects a state of significant physiological derangement consistent with sepsis, in which the likelihood of intensive care admission and death increases. In our cohort, more than half of the patients with qSOFA ≥ 2 required intensive care, and their mortality rates were markedly elevated. In the univariate analysis, altered mental status alone also appeared as a strong risk factor. Confusion constitutes one of the qSOFA criteria and is considered a marker of organ dysfunction in sepsis. The observation that two-thirds of patients with altered mental status died can be interpreted as an indicator of the severity of the septic process. Therefore, in hemodialysis patients present with bacteremia, the presence of altered consciousness, hypotension, or tachypnea should prompt immediate aggressive management and urgent consideration of intensive care support. This finding suggests that qSOFA may be a useful tool for early triage and risk stratification in patients with CRBSI; however, previous studies have shown that its sensitivity may be reduced in individuals with chronic kidney disease and those undergoing hemodialysis [34]. Although qSOFA emerged as an independent predictor in our study, its prognostic performance in this population should be interpreted with caution.

This study has several limitations. Its single-center design and limited sample size may restrict the generalizability of the findings and reduce the statistical power to detect certain associations. Only in-hospital mortality was assessed, and post-discharge outcomes could not be captured. Disease severity was evaluated solely using the qSOFA score; more comprehensive scoring systems or biomarkers were not examined. In addition, antimicrobial lock solutions and catheter prophylaxis protocols are not routinely used in our center, preventing the evaluation of these variables. Variations in hemodialysis unit practices—such as catheter care procedures, choice of antiseptics, use of antimicrobial lock solutions, and infection control strategies—may influence CRBSI incidence and mortality, thereby limiting the generalizability of our results. These considerations highlight the need for larger, multi-center studies employing standardized catheter care protocols to more clearly define prognostic determinants in hemodialysis patients with CRBSI.

On the other hand, a strength of this study is its prospective design with real-time data collection, which enhances the accuracy and completeness of the data. Furthermore, we applied strict diagnostic criteria and included only confirmed CRBSI episodes, which improves the validity of our analysis by focusing on a well-defined patient group. These aspects lend confidence to our findings on the key risk factors for mortality in this high-risk population.

## 5. Conclusions

Catheter-related bloodstream infections in hemodialysis patients are associated with a considerable rate of in-hospital mortality. In this prospective single-center study, we identified high-risk subgroups among HD patients who developed catheter-related infections—specifically those infected with resistant pathogens, presenting with elevated qSOFA scores, or harboring multiple infectious foci. Recognizing these patients early and providing intensive monitoring and prompt, targeted therapeutic interventions may play a critical role in reducing mortality. Future prospective, large-scale multicenter studies are warranted to better characterize prognostic factors in this population and to validate our findings.

## Figures and Tables

**Table 1:** Demographic characteristics, comorbidities, and treatment histories of patients stratified by survival status.

Variables	Survivor	Non-Survivor	*p*
Age, median (IQR)	63 (57–74)	64 (60–83)	0.330
Gender, n (%)			0.942
Male	29 (56.9)	5 (55.6)	
Female	22 (43.1)	4 (44.4)	
Comorbidities, n (%)			
Diabetes mellitus	22 (43.1)	6 (66.7)	0.173
Coronary artery disease	18 (35.3)	4 (44.4)	0.431
Congestive heart failure	12 (23.5)	2 (22.2)	0.651
Chronic obstructive pulmonary disease	11 (21.6)	3 (33.3)	0.349
Cerebrovascular event	4 (7.8)	-	0.512
Thyroid diseases	6 (11.8)	2 (22.2)	0.609
Solid organ malignancy	3 (5.9)	2 (22.2)	0.159
Hematological malignancy	-	1 (11.1)	0.150
Presence of endocarditis	5 (9.8)	2 (22.2)	0.281
Number of diseases, median (IQR)	2 (2–3)	3 (2–3)	0.097
Charlson comorbidity index, median (IQR)	4 (3–6)	5 (4–10.5)	0.231
Duration of hemodialysis			0.715
<1 year	17 (33.3)	4 (44.4)	
1–5 years	18 (35.3)	2 (22.2)	
>5 years	16 (31.4)	3 (33.3)	
Previous treatment history, n (%)			
Hospitalization due to previous history of catheter infection	6 (11.8)	2 (22.2)	0.344
Antibiotic use in the previous three months	25 (49.0)	5 (55.6)	0.500
History of peritoneal dialysis	10 (19.6)	1 (11.1)	0.475

**Table 2:** Comparison of baseline clinical characteristics between survivors and non-survivors.

Variables	Survivor	Non-Survivor	*p*
Initial clinical findings, n (%)			
Rash around the catheter (>2 cm)	14 (27.5)	1 (11.1)	0.279
Purulent discharge around the catheter	10 (19.6)	-	0.169
Catheter dysfunction	16 (31.4)	5 (55.6)	0.153
Nausea-vomiting	48 (94.1)	8 (88.9)	0.488
Confusion	9 (17.6)	6 (66.7)	0.005
Initial vital findings, n (%)			
Fever (≥38.3 °C)	37 (72.5)	3 (33.3)	0.052
Hypoxia (SO_2_ < 93%)	21 (41.2)	5 (55.6)	0.328
Hypotension (<90/60 mm/Hg)	16 (31.4)	4 (44.4)	0.342
Tachypnea (>30 breaths/min)	10 (19.6)	-	0.169
Tachycardia (>100 beats/min)	17 (33.3)	3 (33.3)	0.658
qSOFA ≥ 2	10 (19.6)	5 (55.6)	0.022
Other focus of infection, n (%)	8 (15.7)	6 (66.7)	0.003
Pyocystitis	6 (11.8)	5 (55.6)	0.007
Cholangitis	-	1 (11.1)	0.150
Pneumonia	3 (5.9)	1 (11.1)	0.488

**Table 3:** Baseline laboratory parameters stratified by survival status.

Laboratory Tests (IQR)	Survivor	Non-Survivor	*p*
Hemoglobin (g/L)	9.9 (8.4–10.9)	11.1 (8.3–12.65)	0.351
Leukocyte count (10^9^/L)	10.40 (7.45–14.84)	11.29 (7.72–16.53)	0.656
Neutrophil count (10^9^/L)	8.57 (5.72–12.43)	8.26 (6.94–13.94)	0.425
Lymphocyte count (10^9^/L)	0.90 (0.58–1.39)	0.81 (0.38–2.18)	0.893
Platelet count (10^9^/L)	204 (165–240)	193 (102–237)	0.291
CRP (mg/L)	110 (70–206)	203 (122–250)	0.042
Creatinine (mg/dL)	5.20 (3.91–7.80)	4.40 (2.88–9.55)	0.534
Blood urea nitrogen (mg/dL)	47 (34–68)	48 (33–114)	0.501
Aspartate aminotransferase (U/L)	18 (13–27)	41 (16–176)	0.093
Alanine aminotransferase (U/L)	16 (9–25)	36 (10–62)	0.120
Albumin (g/dL)	3.6 (3.3–4.0)	3.5 (2.9–4.3)	0.282
Creatinine (mg/dL)	5.20 (3.91–7.80)	4.40 (2.88–9.55)	0.534
International normalized ratio	1.1 (1.04–1.3)	1.1 (1.09–1.45)	0.709
D-dimer (ng/mL)	3265 (1756–5637)	3171 (2400–20,222)	0.491
Ferritin (ng/mL)	568 (292–1031)	440 (312–997)	0.498
Procalcitonin (ng/mL)	3.7 (1.1–28.0)	9.1 (0.6–100.0)	0.872
Neutrophils/Lymphocyte ratio	9.03 (4.78–17.19)	23.23 (3.75–29.17)	0.569

CRP: C-reactive protein.

**Table 4:** Distribution of catheter- and microorganism-related risk factors stratified by survival status.

Variables	Survivor	Non-Survivor	*p*
Catheter type n (%)			0.005
Tunneled	42 (82.4)	3 (33.3)	
Non-tunneled	9 (17.6)	6 (66.7)	
Catheter site, n (%)			0.650
Subclavian	36 (70.6)	5 (55.6)	
Jugular	3 (5.9)	1 (11.1)	
Femoral	12 (23.5)	3 (33.3)	
Catheter duration			0.354
1–30 days	12 (23.5)	4 (44.4)	
30–180 days	10 (19.6)	2 (22.2)	
>180 days	29 (56.9)	3 (33.3)	
Catheter withdrawn in the first 48 h	10 (19.6)	1 (11.1)	0.475
Microorganism, n (%)			
MDR	19 (37.3)	8 (88.9)	0.005
Polymicrobial	5 (9.8)	1 (11.1)	0.640
*Staphylococcus aureus*	15 (29.4)	3 (33.3)	0.547
Gram staining			0.516
Gram-positive	34 (66.7)	5 (55.6)	
Gram-negative	12 (23.5)	4 (44.4)	
Gram-positive + -negative	4 (7.8)	-	
*Candida* spp.	1 (2.0)	-	

MDR: multidrug-resistant.

**Table 5:** Univariate and multivariate logistic regression analysis.

Variables	Univariate OR (95% CI)	*p*	Multivariate OR (95% CI)	*p*
Microorganisms’ resistance profile, MDR vs. Sensitive strains	13.474 (1.562–116.245)	0.018	14.850 (1.278–172.581)	0.031
qSOFA ≥ 2 vs. qSOFA < 2	5.125 (1.160–22.636)	0.031	8.757 (1.177–65.135)	0.034
Catheter type, Tunnelled vs. Non-tunneled	9.333 (1.958–44.493)	0.005		
Accompanying other focus of infection, Presence of pyocystitis vs. Absence of pyocystitis	10.750 (2.219–52.089)	0.003	13.059 (1.701–100.227)	0.013
Altered consciousness, Presence vs. Absence	9.333 (1.958–44.493)	0.005		
CRP	1.007 (0.999–1.015)	0.075		

CI: Confidence Interval; OR: Odds Ratio; CRP: C-reactive protein.

## Data Availability

Data are available from the corresponding author upon reasonable request.

## References

[1] Lok C.E., Huber T.S., Lee T., Shenoy S., Yevzlin A.S., Abreo K., Allon M., Asif A., Astor B.C., Glickman M.H. (2020). KDOQI Clinical Practice Guideline for Vascular Access: 2019 Update. Am. J. Kidney Dis..

[2] Miller L.M., MacRae J.M., Kiaii M., Clark E., Dipchand C., Kappel J., Lok C., Luscombe R., Moist L., Oliver M. (2016). Hemodialysis Tunneled Catheter Noninfectious Complications. Can. J. Kidney Health Dis..

[3] Nguyen D.B., Shugart A., Lines C., Shah A.B., Edwards J., Pollock D., Sievert D., Patel P.R. (2017). National Healthcare Safety Network (NHSN) Dialysis Event Surveillance Report for 2014. Clin. J. Am. Soc. Nephrol..

[4] Bokhari S.F.H., Iqbal A., Usman S., Mushtaq U., Mukhtar Z., Naseer B. (2025). A comprehensive review of infection risks and management in hemodialysis access sites. Clin. Exp. Nephrol..

[5] Fram D., Taminato M., Ponzio V., Manfredi S.R., Grothe C., Batista R.E.A., Belasco A., Barbosa D. (2014). Risk factors for morbidity and mortality of bloodstream infection in patients undergoing hemodialysis: A nested case–control study. BMC Res. Notes.

[6] Schamroth Pravda M., Maor Y., Brodsky K., Katkov A., Cernes R., Schamroth Pravda N., Tocut M., Zohar I., Soroksky A., Feldman L. (2024). Blood stream Infections in chronic hemodialysis patients—Characteristics and outcomes. BMC Nephrol..

[7] Campos E., Cuevas-Budhart M.A., Cedillo-Flores R., Candelario-López J., Jiménez R., Flores-Almonte A., Ramos-Sanchez A., Divino Filho J.C. (2024). Is central venous catheter in haemodialysis still the main factor of mortality after hospitalization?. BMC Nephrol..

[8] Weldetensae M.K., Weledegebriel M.G., Nigusse A.T., Berhe E., Gebrearegay H. (2023). Catheter-Related Blood Stream Infections and Associated Factors Among Hemodialysis Patients in a Tertiary Care Hospital. Infect. Drug Resist..

[9] Nanyunja D., Chothia M.Y., Opio K.C., Ocama P., Bwanga F., Kiggundu D., Byakika-Kibwika P. (2022). Incidence, microbiological aspects and associated risk factors of catheter-related bloodstream infections in adults on chronic haemodialysis at a tertiary hospital in Uganda. IJID Reg..

[10] Sahli F., Feidjel R., Laalaoui R. (2017). Hemodialysis catheter-related infection: Rates, risk factors and pathogens. J. Infect. Public Health.

[11] Borghese O., Campion M., Magana M., Pisani A., Di Centa I. (2024). Re-hospital admission, morbidity and mortality rate in patients undergoing tunnelled catheter implantation for haemodialysis. J. Med. Vasc..

[12] Yildirim S., Yilmaz B., Yilmaz E., Derici U.B. (2024). Metastatic spondylodiscitis in central venous catheter related bloodstream infections in hemodialysis patients: Risk factors and prognosis. J. Vasc. Access.

[13] Chen C., Ma S., Huang W. (2024). Prognostic risk factors of catheter-related bloodstream infection in patients with maintenance hemodialysis. Zhonghua Wei Zhong Bing Ji Jiu Yi Xue.

[14] Katneni R., Hedayati S.S. (2007). Central venous catheter-related bacteremia in chronic hemodialysis patients: Epidemiology and evidence-based management. Nat. Clin. Pract. Nephrol..

[15] Shahar S., Mustafar R., Kamaruzaman L., Periyasamy P., Pau K.B., Ramli R. (2021). Catheter-Related Bloodstream Infections and Catheter Colonization among Haemodialysis Patients: Prevalence, Risk Factors, and Outcomes. Int. J. Nephrol..

[16] Fysaraki M., Samonis G., Valachis A., Daphnis E., Karageorgopoulos D.E., Falagas M.E., Stylianou K., Kofteridis D.P. (2013). Incidence, clinical, microbiological features and outcome of bloodstream infections in patients undergoing hemodialysis. Int. J. Med. Sci..

[17] Mermel L.A., Allon M., Bouza E., Craven D.E., Flynn P., O’Grady N.P., Raad I.I., Rijnders B.J., Sherertz R.J., Warren D.K. (2009). Clinical practice guidelines for the diagnosis and management of intravascular catheter-related infection: 2009 Update by the Infectious Diseases Society of America. Clin. Infect. Dis..

[18] Evans L., Rhodes A., Alhazzani W., Antonelli M., Coopersmith C.M., French C., Machado F.R., McIntyre L., Ostermann M., Prescott H.C. (2021). Surviving sepsis campaign: International guidelines for management of sepsis and septic shock 2021. Intensive Care Med..

[19] Magiorakos A.P., Srinivasan A., Carey R.B., Carmeli Y., Falagas M.E., Giske C.G., Harbarth S., Hindler J.F., Kahlmeter G., Olsson-Liljequist B. (2012). Multidrug-resistant, extensively drug-resistant and pandrug-resistant bacteria: An international expert proposal for interim standard definitions for acquired resistance. Clin. Microbiol. Infect..

[20] Brownlee H.C., Richards J., Trent N.D. (2025). Pyocystis.

[21] Nelveg-Kristensen K.E., Laier G.H., Heaf J.G. (2018). Risk of death after first-time blood stream infection in incident dialysis patients with specific consideration on vascular access and comorbidity. BMC Infect. Dis..

[22] Phillips J., Chan D.T., Chakera A., Swaminathan R., Patankar K., Boudville N., Lim W.H. (2023). Haemodialysis vascular catheter-related blood stream infection: Organism types and clinical outcomes. Nephrology.

[23] Almenara-Tejederas M., Rodríguez-Pérez M.A., Moyano-Franco M.J., de Cueto-López M., Rodríguez-Baño J., Salgueira-Lazo M. (2023). Tunneled catheter-related bacteremia in hemodialysis patients: Incidence, risk factors and outcomes. A 14-year observational study. J. Nephrol..

[24] Hu M., Chua S.L. (2025). Antibiotic-Resistant Pseudomonas aeruginosa: Current Challenges and Emerging Alternative Therapies. Microorganisms.

[25] Surapat B., Montakantikul P., Malathum K., Kiertiburanakul S., Santanirand P., Chindavijak B. (2020). Microbial epidemiology and risk factors for relapse in gram-negative bacteria catheter-related bloodstream infection with a pilot prospective study in patients with catheter removal receiving short-duration of antibiotic therapy. BMC Infect. Dis..

[26] El Khudari H., Ozen M., Kowalczyk B., Bassuner J., Almehmi A. (2022). Hemodialysis Catheters: Update on Types, Outcomes, Designs and Complications. Semin. Interv. Radiol..

[27] Kadhem N.S., Atya A.K. (2025). The growing concern of MDR/XDR bacteria in patients undergoing dialysis: A cross-sectional study. KIDNEYS.

[28] Manhal F.S., Mohammed A.A., Ali K.H. (2012). Urinary tract infection in hemodialysis patients with renal failure. J. Fac. Med. Baghdad.

[29] Scherberich J.E., Fünfstück R., Naber K.G. (2021). Urinary tract infections in patients with renal insufficiency and dialysis—Epidemiology, pathogenesis, clinical symptoms, diagnosis and treatment. GMS Infect. Dis..

[30] Prabhahar A., Vijaykumar N.A., Selvam S., Ramchandran R., Sethi J., Pannu A.K., Sharma N. (2024). Characteristics and Prognosis of Infectious Disease Emergencies in Patients with Chronic Kidney Disease in India. Indian. J. Crit. Care Med..

[31] Jaber B.L. (2005). Bacterial infections in hemodialysis patients: Pathogenesis and prevention. Kidney Int..

[32] Perman S.M., Mikkelsen M.E., Goyal M., Ginde A., Bhardwaj A., Drumheller B., Sante S.C., Agarwal A.K., Gaieski D.F. (2020). The sensitivity of qSOFA calculated at triage and during emergency department treatment to rapidly identify sepsis patients. Sci. Rep..

[33] Seymour C.W., Liu V.X., Iwashyna T.J., Brunkhorst F.M., Rea T.D., Scherag A., Rubenfeld G., Kahn J.M., Shankar-Hari M., Singer M. (2016). Assessment of Clinical Criteria for Sepsis: For the Third International Consensus Definitions for Sepsis and Septic Shock (Sepsis-3). JAMA.

[34] Nishiwaki H., Sasaki S., Hasegawa T., Sasai F., Kawarazaki H., Minatoguchi S., Uchida D., Koitabashi K., Ozeki T., Koiwa F. (2019). External validation of the quick Sequential Organ Failure Assessment score for mortality and bacteraemia risk evaluation in Japanese patients undergoing haemodialysis: A retrospective multicentre cohort study. BMJ Open.

